# Evaluating neural encoding of prosody-related F0 changes in emotional speech using the speech FFR in normal-hearing adults

**DOI:** 10.1038/s41598-026-50121-0

**Published:** 2026-04-29

**Authors:** Maryam Karimi-Boroujeni, Sajad Sadeghkhani, Christian Giguère, Saeid R. Seydnejad, Hilmi R. Dajani

**Affiliations:** 1 School of Rehabilitation Sciences, University of Ottawa, Ottawa, ON Canada; 2 School of Electrical Engineering and Computer Science, University of Ottawa, Ottawa, ON Canada; 3 Department of Electrical Engineering, Shahid Bahonar University of Kerman, Kerman, Iran

**Keywords:** Prosody processing, Emotional speech, Frequency following response (FFR), Neuroscience, Psychology, Psychology

## Abstract

Emotional information in speech is conveyed through prosodic cues, including variations in fundamental frequency (F0 contour), intensity, and duration. Emotion perception has been largely studied behaviorally but the neural mechanisms underlying it are not well understood. The Speech-evoked Frequency Following Response (FFR) is a non-invasive neural measure reflecting the encoding of speech acoustics in the auditory system. This study investigated the extent to which the FFR can represent prosody-related F0 contours and compared neural responses between male and female listeners. Sixteen normal-hearing adults underwent FFR recording in response to the word “balloon” spoken with sad and happy emotion by a male and a female talker. Using a pitch-tracking algorithm, F0 tracking accuracy was quantified via Root Mean Squared Error (RMSE) and an accuracy percentage. The results showed that the FFR can track emotional F0 contours; however, the degree of accuracy is modulated by the acoustic characteristics of emotions and talker’s voice. Sad emotional speech and the male talker voice were associated with enhanced F0 tracking, consistent with their acoustic features. On the other hand, F0 tracking accuracy did not differ by listener sex. These findings provide new insights into the use of FFR as a neural measure for prosody assessment.

**Supplementary Information:**

The online version contains supplementary material available at 10.1038/s41598-026-50121-0.

## Introduction

The speech signal is the primary means of conveying information in interactive human communication^1,2^. Through the vocal apparatus, a sequence of basic linguistic units is produced in acoustical form; syllables build meaningful words, and then words are arranged into phrases to state more complex concepts^3,4^. In natural speech, these segments are not uttered at a fixed tone, loudness, and rate. The segments are threaded together with different melodies and rhythms, which constitute the suprasegmental features of speech^5,6^. Some speech perception studies place less emphasis on the suprasegmental information, even though it is an important contributor of information that complements the properties of segmental sounds^7^.

Suprasegmental information, also called prosody, contributes to conveying the emotional state of the speaker through which the listener can understand what the speaker feels and thinks^8–10^. The emotional function of prosody is defined acoustically as variations in fundamental frequency (F0), intensity, and duration^11^. To perceive emotional patterns in speech, these acoustic-based prosodic cues are initially received and encoded by the peripheral auditory system^9,12^. Thereafter, the emotion-related cues are transmitted through the ascending auditory pathway, including subcortical and cortical structures, to undergo further processing^13,14^. Additionally, cognitive top-down mechanisms are engaged in a feedback information flow to extract and deduce contextual meaning and pick out pertinent information^12,15^. Despite the vast neural networks involved in prosody processing, most studies have focused on the behavioral aspects of emotion perception, and the neural processing of acoustically defined features of prosodic speech remains poorly understood in both normal-hearing individuals and those with impairment.

The speech-evoked Frequency Following Response (FFR), recorded via scalp electrodes, is a non-invasive neural measure reflecting the temporal and spectral encoding of speech in the central auditory system^16,17^. The FFR arises from synchronized neural activity and primarily provides a neural representation of pitch-related aspects of the stimulus within the auditory pathway^16,18^. Although many FFR studies have relied on averaged measures of F0 amplitude, some studies have shown that the phase-locked neural activity underlying the FFR is sufficiently dynamic to reliably track small variations in speech periodicity over short time scales such as tonal sweeps, synthetic/natural vowels, and isolated syllables^19–21^. However, it remains unclear whether the FFR can successfully capture larger F0 variations over longer time windows extending beyond segmental information, as observed in emotional prosody. In addition, neural processing with more natural, meaningful speech may reflect top-down influences, and differ from that associated with simplified stimuli such as isolated syllables^22^. This study focused on a range of distinct prosodic F0 contours using a natural two-syllable word spoken with sad and happy emotions. Because male and female voices differ in key acoustic features (e.g., mean F0, rate of F0 change, and harmonic structure) that influence FFR pitch encoding^23^, both talkers were included to provide acoustically diverse stimuli and to assess whether FFR tracking is maintained across natural variations in voice characteristics. Therefore, as a proof of concept, the study aimed to investigate whether the FFR can track prosodic F0 contours in natural speech across different emotion and talker conditions.

Evidence also suggests that F0 representation in the FFR may vary with listener sex, though results have been inconsistent. While stronger F0 amplitude has been reported in female listeners for short stimuli with fixed F0^24^, often linked to anatomical and physiological differences in auditory encoding, other studies have shown comparable F0 phase-locking across sexes for stimuli with fixed and time-varying F0^25,26^. Whether such listener sex effects extend to tracking of prosody-related F0 changes over time remains unclear. As an exploratory objective, we also examined potential listener-sex differences in the neural tracking of dynamic F0 changes in emotional speech. The suprasegmental nature of the present stimuli may offer supplementary insight into sex differences in the neural encoding of speech prosody.

Using electrophysiological data, this study advances understanding of how efficiently a healthy auditory system can use prosodic features in natural speech processing.

## Methods and materials

### Participants

A total of 16 adults (8 males, 8 females), defined by self-reported biological sex, between 18 and 31 years of age participated in this study. All participants had symmetric normal hearing defined as pure-tone audiometric thresholds ≤ 25 dB HL at octave frequencies between 250 and 8000 Hz in both ears. The four-frequency pure-tone average (PTA_500−4000_) ranged from − 4 to 19 dB HL in the right ear and from − 6 to 20 dB HL in the left ear across individuals. Otoscopy confirmed no abnormalities in the external auditory canal and tympanic membrane in any participant. No participant had a history of neurological diseases, brain injuries, or psychiatric problems, as screened through a general development questionnaire. All participants were native or near-native English speakers who scored 9 or 10 on a 10-point scale in self-report questions from the Language Experience and Proficiency Questionnaire (LEAP-Q)^27^ about their proficiency in speaking and understanding. Given that speakers of tonal languages can be more sensitive to F0 variations^28,29^, only individuals without a tonal language background were recruited. Additionally, since previous studies have shown that musicians exhibit enhanced FFR^30,31^, the Demographic and Music Experience Questionnaire^32^ was used to ensure that only non-musicians were included. To exclude the negative effect of cognitive impairment in prosody perception, all participants were screened using the Standardized Mini-Mental State Examination (SMMSE)^33^ to assess five cognitive domains, including orientation, registration, attention and calculation, recall, and language, with inclusion based on scoring 29 or 30 on a scale of 30. Written informed consent was required from all, with experimental protocols approved by the Office of Research Ethics and Integrity at the University of Ottawa under ethics file number H-03-22-7915. All methods were carried out in accordance with the ethical principles outlined in the Declaration of Helsinki.

### Stimuli

**Fig. 1:**
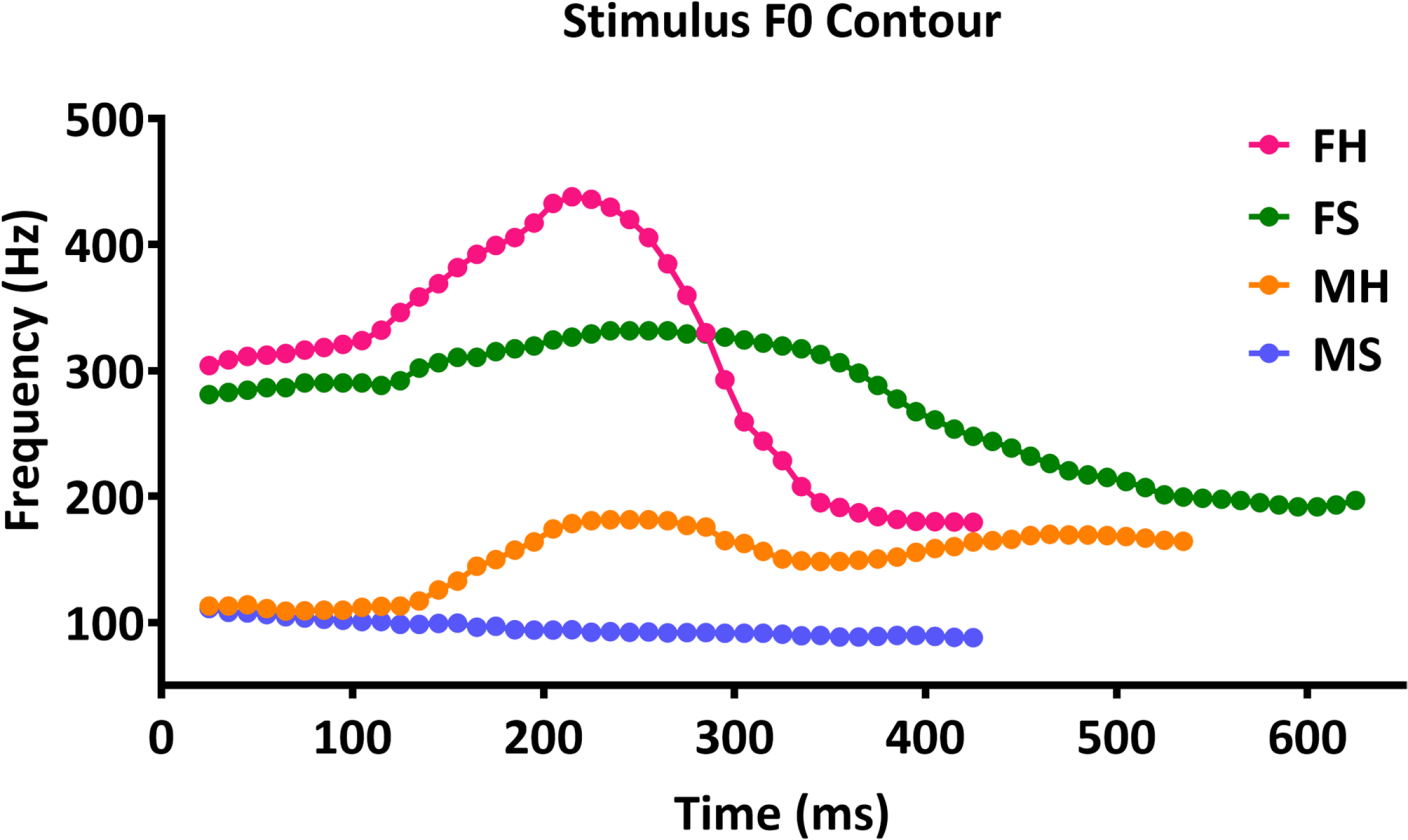
F0 contours for the stimulus word “balloon” spoken by a male and a female with two emotions, sad and happy. The blue curve represents the male talker with sad emotion (MS), the orange curve the male talker with happy emotion (MH), the green curve the female talker with sad emotion (FS), and the pink curve the female talker with happy emotion (FH).

The word “balloon” uttered by a male and a female talker with sad and happy emotions was used to elicit the FFR. This word was extracted from the sentence “*No*,* I burst the balloon!*” in the emotional speech database (ESD)^34^. The ESD database contains 350 parallel utterances spoken by 10 native English and 10 native Chinese speakers across 5 emotions (neutral, happy, angry, sad, and surprise). The word “balloon” was chosen for its fully voiced bisyllabic structure, making it well-suited for tracking suprasegmental variations in F0 across the entire stimulus. This study targeted sad and happy emotions produced by one male English speaker (Speaker 11) and one female English speaker (Speaker 15), selected for their distinct F0 contours, including flat, rising, falling, and rising-falling contours. Figure 1 illustrates the F0 variations for the word “balloon” across emotional contexts for both male and female talkers. The duration of the stimuli also varied across conditions, with the male sad stimulus lasting 457 ms, male happy 565 ms, female sad 655 ms, and female happy 450 ms. Table 1 summarizes key F0 parameters for each condition, including mean, maximum, minimum, and range values. Higher mean F0, broader F0 ranges, and greater rates of F0 change were observed for happy compared to sad emotions, with female talker showing mostly higher F0 values than male talker. F0 parameters were extracted using Praat (version 6.2.05; www.praat.org) with the default settings.

**Table 1:** List of acoustic parameters related to F0 across all the conditions examined.

Word “balloon”		Mean F0 (Hz)	Max F0 (Hz)	Min F0 (Hz)	F0 range (Hz)
Talker	Emotion				
Male	Sad	95.5	107.8	88.1	19.7
Male	Happy	150.9	182.5	108.1	74.4
Female	Sad	272.5	332.2	189.6	142.6
Female	Happy	312.9	432.9	179.1	253.8

### FFR recording

The FFR responses were recorded with the Duet 2-channel AEP system (Intelligent Hearing Systems, Miami, FL) using four Ag/AgCI disposable disc electrodes. In accordance with the International Electrode System IES 10–20 norms, two inverting electrodes were placed at A1 and A2 (right and left earlobes), a noninverting electrode was positioned at Cz (vertex), and the ground electrode was located at Fz (forehead). Prior to electrode placement, the skin at these sites was prepared using an alcohol wipe and Nuprep Skin Prep Gel. The electrode impedances were kept below 5 kΩ, and inter-electrode impedances did not exceed 3 kΩ.

Two-channel FFR recordings were made over a time window of 716.8 ms (including a 40 ms pre-stimulus time), with an online filter setting of 30–1500 Hz, and sampling period of 175 $\:\mu\:$s. The four stimulus conditions, consisting of the spoken word /balloon/ by one male and one female speaker expressing happy and sad emotions, were presented binaurally through a shielded insert earphone at 80 dB SPL and a rate of 1.26 sweeps per second with an alternating polarity. Individual sweeps were automatically rejected as artifacts during the acquisition if they exceeded 31 microvolts after stimulus onset. This experiment was conducted in two sessions on different days, each lasting a maximum of three hours. Each session included 1500 sweeps (three blocks of 500) of each of the four conditions, resulting in 12 blocks of recording overall. After completing 6 blocks in a session, participants were given a 15-minute break to reduce fatigue. In total, 3000 artifact free responses (6 blocks of 500) were acquired per condition for each participant. To prevent order effects, the sequence of emotions and speakers was randomized across participants using a complete counterbalancing approach, ensuring that no emotion or speaker was repeatedly presented in the same order.

The experiment was conducted in an electrically shielded and soundproof booth where participants were seated comfortably in a reclining chair. To minimize artifacts, participants were encouraged to close their eyes, relax, and limit body movements during the recording. They were also asked to try not to fall asleep.

### Data analysis

For each condition by participant, the 3000 response sweeps were coherently averaged. To account for neural delays in the FFR, the stimulus waveform was shifted forward by 10 ms. To extract the F0 contour, both the stimulus and averaged response were segmented into 50 ms frames with a 40ms overlap. The stimulus F0 contour was captured by computing the median of F0 values obtained from each frame using speech pitch estimation methods in MATLAB 2024a specifically the ‘pitch’ and ‘pitchnn’ commands. To extract the response F0 value we followed a Harmonic Amplitude Summation (HAS) approach^35^. In this approach, the magnitude spectrum of each response frame was computed using the Fast Fourier Transform (FFT). Then, a ± 50 Hz frequency search range with 1 Hz increments was defined around the stimulus F0 of the corresponding time-aligned frame. For each candidate frequency within this range, a harmonic signal with a fundamental frequency equal to the candidate value was generated. The number of harmonics in the generated signal was set to 4 for the sad male condition, and to 2 for the remaining conditions, as responses in the sad male condition exhibited a clearer harmonic structure. Subsequently, its magnitude spectrum was computed following the same procedure used for the response. These two magnitude spectra were then element-wise multiplied and summed. The summed values for all candidate frequencies form a one-dimensional contour over the search range. The candidate corresponding to the most prominent peak in this contour was selected as the response F0. Further details on this approach are found in^34^.

Two evaluation metrics were used to assess the accuracy of F0 estimation: (1) Root-Mean-Square Error (RMSE) which quantifies the average deviation between the estimated response and reference stimulus F0 contours, and (2) 5% accuracy which quantifies the proportion of response frames where the estimated F0 values fall within 5% of their corresponding stimulus F0 values^36^. The 5% accuracy criterion is much smaller than the F0 variations relative to the mean F0 in our four stimuli (Table 1). Together, these metrics respectively capture both absolute tracking error and proportional pitch-tracking accuracy across stimuli with different F0 ranges (e.g., male and female voices).

### Statistical analysis

F0 tracking measures (RMSE and 5% accuracy) were analyzed using two-way repeated-measures analysis of variance (RM-ANOVA) with emotion (sad, happy) and talker sex (male, female) as within-subject factors. Follow-up comparisons were conducted as planned simple effects within the ANOVA framework using Fisher’s LSD test. Because each within-subject factor comprised two levels, the sphericity assumption was satisfied, and no Greenhouse-Geisser correction was applied. To explore listener sex differences (male vs. female) in the neural processing of emotional F0 contours, exploratory analyses were conducted using unpaired two-tailed t-tests performed separately for each F0 tracking metric, with Welch’s correction applied when variances were unequal. No additional p-value adjustment was applied to these secondary analyses. All statistical analyses and data visualization were performed using GraphPad Prism 10.0. Results with *p-values* < 0.05 were considered statistically significant.

## Results

### Spectrographic representation and F0 contour of the FFR

**Fig. 2:**
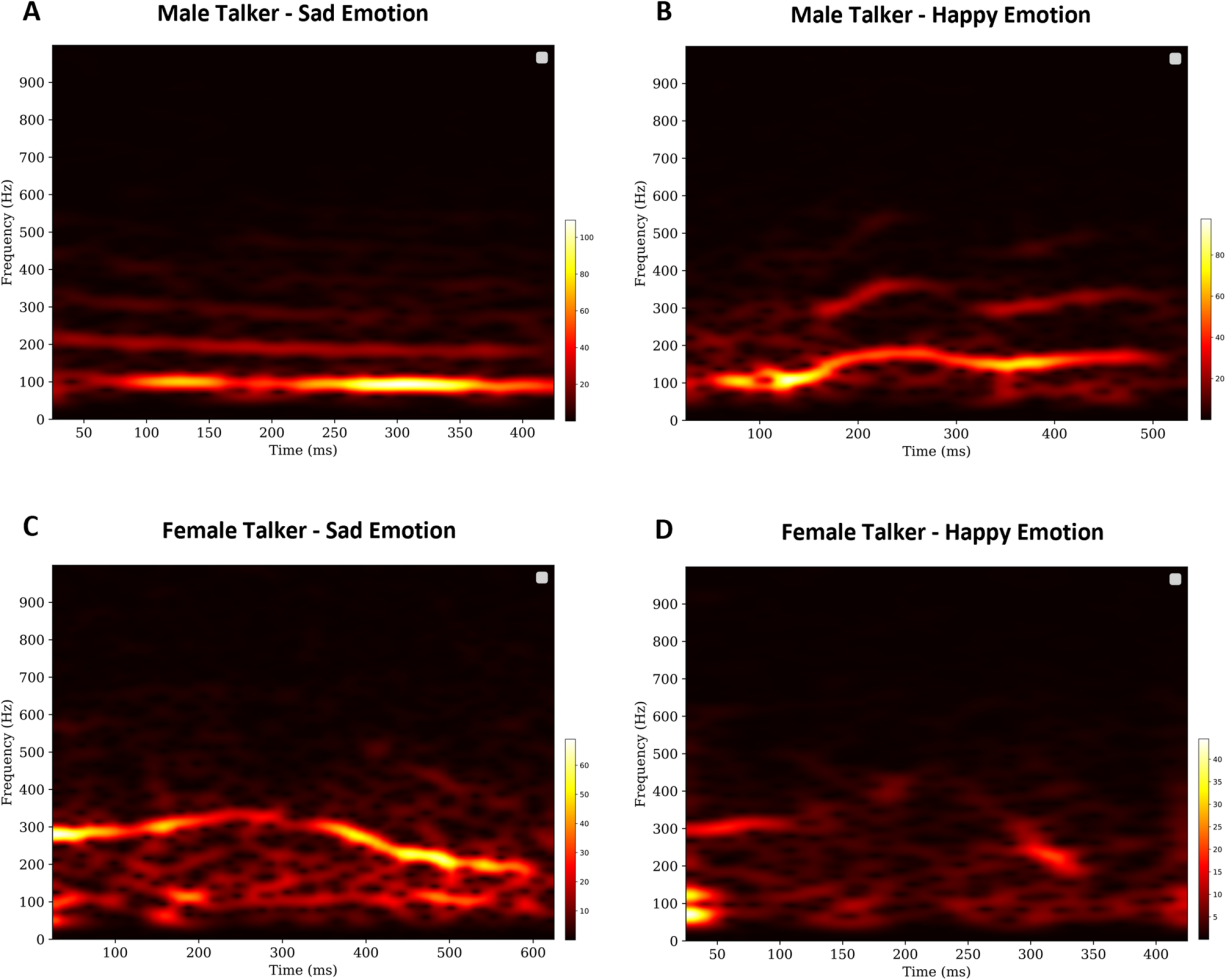
Spectrograms of the average response across all participants (*n* = 16) for the four experimental conditions: male talker and sad emotion (**A**), male talker and happy emotion (**B**), female talker and sad emotion (**C**), and female talker and happy emotion (**D**).

To ensure the robustness of the recorded responses, aggregate spectrograms (6 blocks × 16 participants) were examined for each condition before F0 extraction (Fig. 2). The spectrograms were generated with a window length of 50 ms and an overlap of 40 ms. In the response spectrograms for stimuli presented in the male voice (Fig. 2A–B), strong energy was observed at F0, 2F0, and 3F0, whereas female-voiced stimuli (Fig. 2C–D) showed less prominent F0 and very attenuated harmonics. These observations highlight potential differences in the neural encoding of F0 as a function of stimulus characterisitics.

**Fig. 3:**
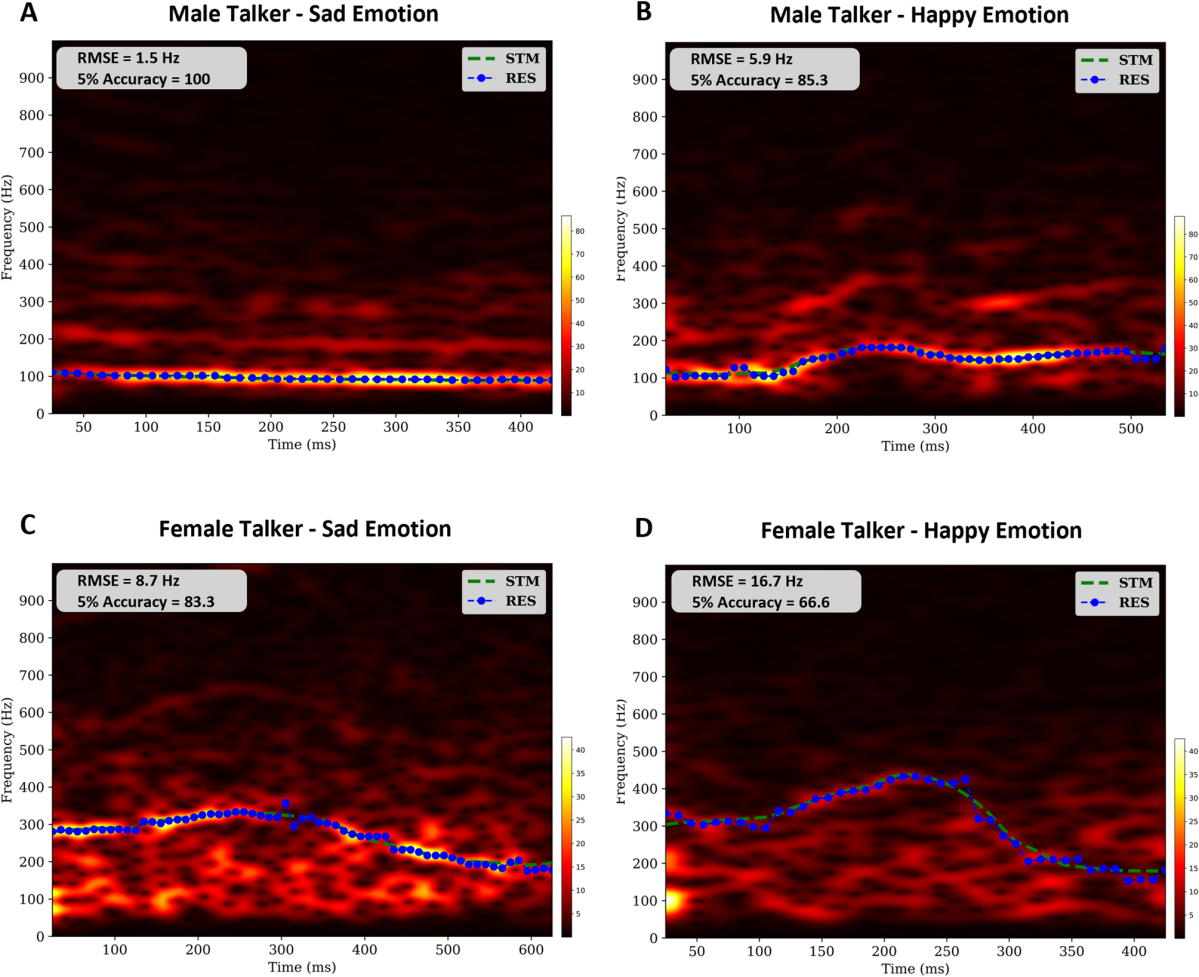
Spectrograms of Participant 13, showing overlaid F0 contours extracted from the stimulus waveform (green curves) and evoked FFR (blue curves) in response to the word “balloon” spoken by male talker and sad emotion (**A**), male talker and happy emotion (**B**), female talker and sad emotion (**C**), and female talker and happy emotion (**D**).

The HAS algorithm was subsequently applied to each participant’s FFR to extract the response F0 contour, and RMSE and 5% accuracy were then computed. Figure 3 displays spectrograms from a selected participant (Participant 13), illustrating that the extracted response F0 contour tracks the stimulus F0 across all four conditions, although its accuracy varies across conditions. According to the 5% accuracy and RMSE metrics for this participant, sad male condition resulted in highest accuracy and lowest error, followed by happy male, sad female, and happy female. Spectrograms with superimposed F0 contours for each participant across all conditions are available in Supplementary Fig. 1. Table 2 summarizes the mean RMSE and 5% accuracy for each of the four stimulus conditions across the 16 participants.

**Table 2:** Mean F0 tracking performance across the four stimulus conditions (*N* = 16).

Stimulus condition		Mean RMSE (Hz)	Mean 5% accuracy (%)
Talker	Emotion		
Male	Sad	2.4	96.9
Male	Happy	7.5	81.9
Female	Sad	12.0	81.8
Female	Happy	17.6	63.8

### Differences in FFR F0 tracking accuracy across stimuli

**Fig. 4:**
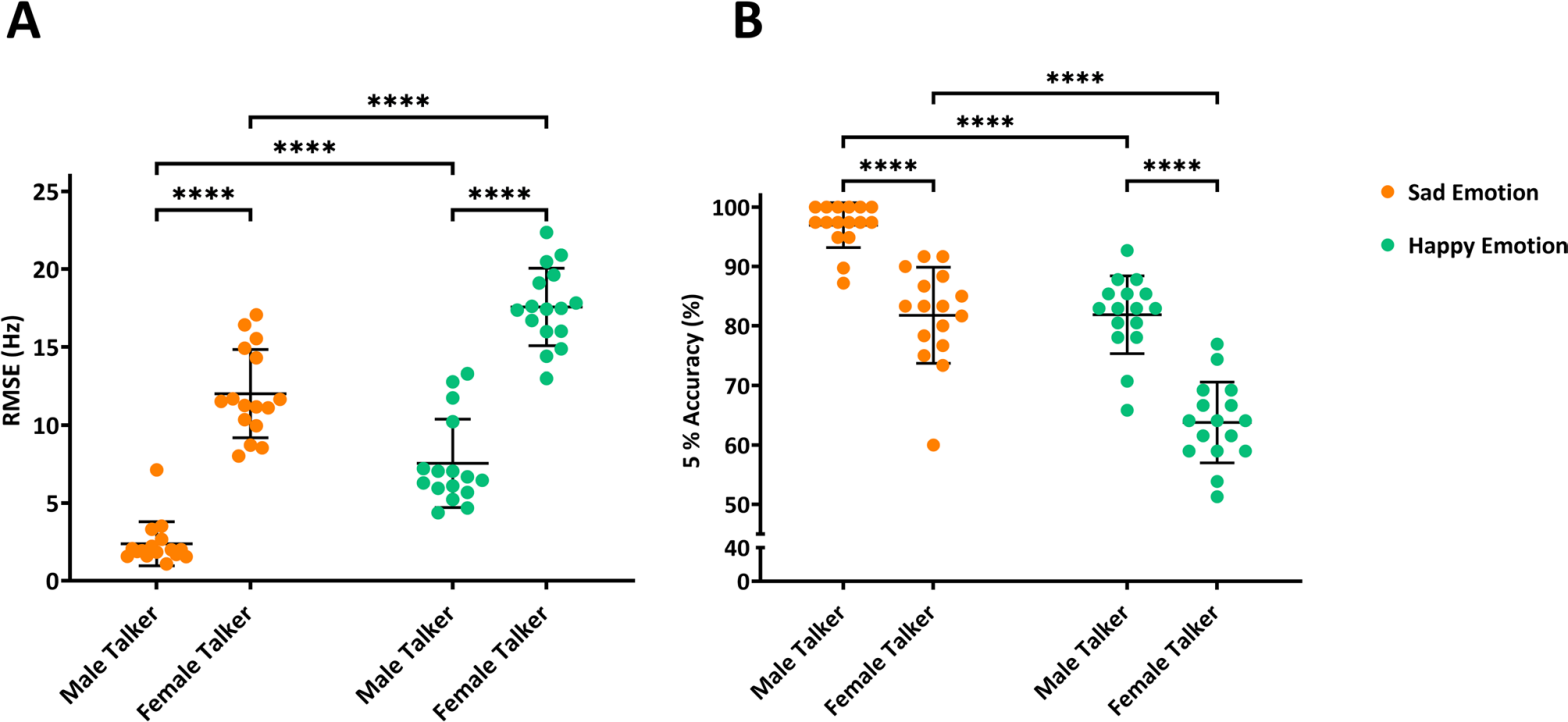
FFR F0 tracking accuracy, measured by either RMSE (**A**) or 5% accuracy (**B**) across all participants (*n* = 16), in response to the word “balloon” spoken by male and female talkers with sad and happy emotions. Data are grouped by talker sex along the x-axis, with emotional condition indicated by color (sad: orange; happy: green). Each dot represents an individual participant, and horizontal bars show the mean ± SD. Statistical significance is shown as follows: * *p* < 0.05, ** *p* < 0.01, *** *p* < 0.001, and **** *p* < 0.0001.

Figure 4 illustrates the results of two-way repeated-measures ANOVAs assessing the effects of emotion (sad, happy) and talker sex (male, female) on 5% accuracy and RMSE across participants.

As shown in Fig. 4A, for RMSE, the analysis revealed a significant main effect of emotion type (F(1, 15) = 124.7, *p* < 0.0001, η²*p* = 0.89), and a significant main effect of talker (F(1, 15) = 276.1, *p* < 0.0001, η²*p* = 0.95), with no emotion × talker interaction (F(1,15) = 0.1, *p* = 0.74). Follow-up simple effects tests showed significantly lower RMSE for sad compared with happy stimuli for both male (mean difference = 5.2 Hz, *p* < 0.0001) and female talkers (mean difference = 5.6 Hz, *p* < 0.0001). Additionally, RMSE was significantly lower for male than for female talkers across both sad (mean difference = 9.6 Hz, *p* < 0.0001) and happy stimuli (mean difference = 10.0 Hz, *p* < 0.0001).

Consistent with the RMSE findings, 5% accuracy (Fig. 4B) showed significant main effects of emotion type (F(1,15) = 133.3, *p* < 0.0001, η²*p* = 0.90) and talker sex (F(1,15) = 103.1, *p* < 0.0001, η²*p* = 0.87) in the two-way repeated-measures ANOVA, without a significant emotion × talker interaction (F(1,15) = 1.16, *p* = 0.30). Follow-up simple effects analyses showed that sad stimuli were encoded with higher 5% accuracy than happy stimuli for both male (mean difference = 15.1%, *p* < 0.0001) and female talkers (mean difference = 18.0%, *p* < 0.0001). Moreover, male talker exhibited higher accuracy than female talker for both sad (mean difference = 15.2%, *p* < 0.0001) and happy emotion (mean difference = 18.1%, *p* < 0.0001).

### Listener sex differences in FFR F0 tracking accuracy

Differences in FFR F0 tracking between male and female listeners across stimulus conditions were evaluated using independent two-tailed t-tests for RMSE and 5% accuracy, applying Welch’s correction when necessary.

**Fig. 5:**
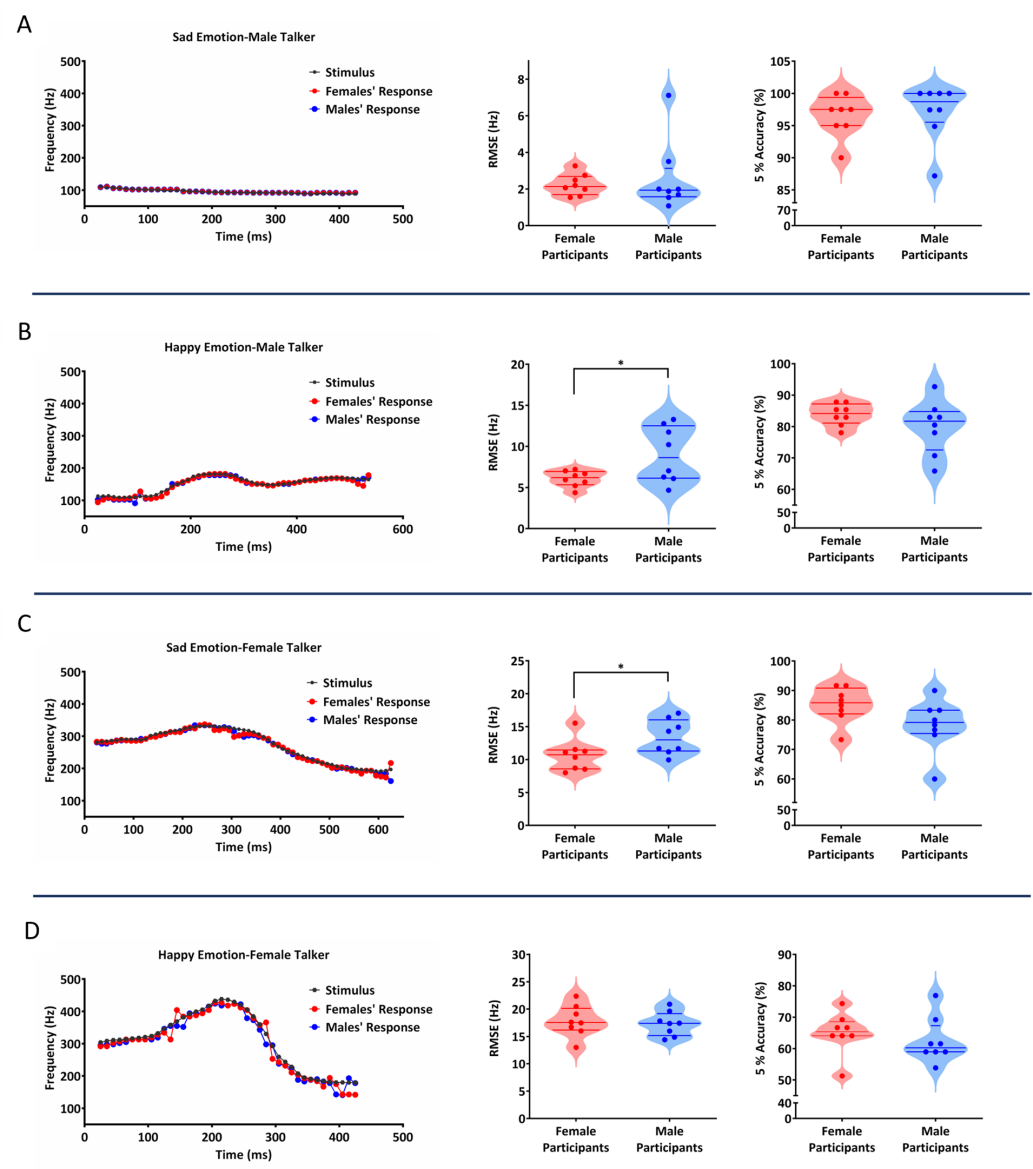
FFR F0 tracking accuracy as a function of listener sex, shown using RMSE (middle panel) and 5% accuracy (right panel). F0 contours extracted (left panel) from the stimulus waveform (black curves) and evoked FFR according to the listener sex, with female participants (red curves) vs. male participants (blue curves) in normal hearing individuals in response to the word “balloon” spoken by male talker and sad emotion (**A**), male talker and happy emotion (**B**), female talker and sad emotion (**C**), and female talker and happy emotion (**D**).

As illustrated in Fig. 5 (left panels), the male- and female-averaged FFR F0 contours and response F0 contours were highly similar across conditions. Consistent with this observation, statistical analyses indicated no significant listener sex differences in either RMSE or 5% accuracy for the sad male (RMSE: mean difference = 0.3 Hz, *p* = 0.63; 5% accuracy: mean difference = 0.5%, *p* = 0.78) and happy female conditions (RMSE: mean difference = 0.5 Hz, *p* = 0.68; 5% accuracy: mean difference = 2.5%, *p* = 0.47; Fig. 5A and D). In the happy male condition, RMSE differed significantly between male and female listeners (mean difference = 3.0 Hz, *p* = 0.0451), with female listeners showing lower RMSE values, whereas 5% accuracy did not differ between groups (mean difference = 4.0%, *p* = 0.25; Fig. 5B). Similarly, in the sad female condition, no significant listener sex difference was observed for 5% accuracy (mean difference = 6.9%, *p* = 0.08), although RMSE was significantly lower in female than in male listeners (mean difference = 2.7 Hz, *p* = 0.0455; Fig. 5C).

## Discussion

F0 variations over time, extending beyond single speech segments, create different prosodic patterns that can convey various emotional states during a conversation. Using FFR recordings from 16 individuals with normal hearing, we investigated the applicability of the FFR in assessing the neural encoding of time-varying prosodic F0 contours.

Our results showed that the FFR can preserve pitch-related information in response to a spoken word token by a male and a female voice with either sad or happy emotions, suggesting that auditory neural circuits can dynamically phase lock to time-variant F0 in emotional speech. The accuracy of FFR F0 tracking is supported by the relationship between the stimulus acoustics and the obtained results. Comparing Tables 1 and 2, the mean RMSE for tracking F0 is much smaller than the F0 changes in the stimuli. For the least challenging condition (male talker-sad emotion), the mean RMSE at 2.4 Hz is much smaller than the F0 range of 19.7 Hz in the stimulus. In the most challenging condition (female talker-happy emotion), the mean RMSE increases to 17.6 Hz but remains small relative to the F0 range of 253.8 Hz in the stimulus. Similarly, the results obtained with the 5% accuracy criterion indicated good tracking performance relative to the stimulus F0 variations. Furthermore, as shown in the individual results in the supplementary materials and in Fig. 4, good tracking accuracy was also achieved across all participants in all four stimuli. Although previous studies demonstrated that the FFR can track dynamic pitch contours at the segmental level^19,21^, our findings extend this evidence to suprasegmental variations associated with emotional expression over longer-duration speech stimuli.

When considering emotion exclusively, we found that sad stimulus elicited more robust FFR F0 tracking than happy stimulus for both male and female talkers, as reflected by higher 5% accuracy and lower RMSE. In this experiment, because participants listened to a single repeated token extracted from emotional sentences, differences in F0 tracking likely reflect only the prosody-related acoustic characteristics of the talkers, not the emotional state of the listeners. Sadness, as shown in Table 1; Fig. 1, is typically characterized by a lower average F0 height and a falling F0 trajectory with lower rate of F0 change compared to the happy stimulus^37–39^. Such acoustic properties are expected to influence the fidelity of neural phase locking and therefore affect FFR pitch tracking. Pitch, as a complex perceptual attribute, is shaped by different characteristics, including average F0 height, direction of F0 movement, and magnitude of F0 slope^40,41^. In the context of FFR recordings, previous studies have consistently observed that F0 response magnitude decreases as pitch height of the stimulus increases^16,42,43^. Other studies focusing on pitch direction have demonstrated that FFR pitch contours can follow rising and falling trajectories in synthetic Mandarin tones or tone glides, with some reporting stronger responses for rising contours^19^ and others reporting comparable pitch-tracking accuracy^44,45^. This discrepancy was explained by the idea that pitch direction alone does not fully account for FFR behavior^45^. The rate and shape of F0 change have been shown to play an important role in neural tracking, with slower and shallower contours tending to support more robust phase-locking^43,45,46^. This suggests there is a complex relationship between pitch representation and stimulus frequency content and periodicity. Because our stimuli contained nonlinear, continuously varying F0 patterns over longer time windows, they more closely resemble real speech for the neural tracking of emotion-related F0 contours in the auditory system. Consistent with prior studies, the present findings indicate that the neural tracking indexed by the FFR was likely sensitive to F0-related acoustic cues rather than to emotion-specific processing.

In addition to emotion type, F0 tracking in the FFR appeared to vary with the acoustic characteristics of male- and female-spoken voices. Across both emotional conditions, the male talker produced more accurate F0 tracking rather than female talker. The male voice in our study had an F0 nearly half that of the female voice (Table 1), which likely explains the stronger FFR tracking for the male talker and suggests an effect of voice acoustics rather than talker gender per se. Consistently, previous studies have reported that speech produced by a talker with a lower F0 elicits larger FFR amplitude^23,47,48^. Supporting this interpretation, Van Canneyt et al. also showed stronger F0 responses for male-narrated stories, which were characterized by a lower and more stable F0 than female narration^49^. Notably, they further indicated that modifying the female speech to reduce the rate of F0 change and enhance higher harmonics increased the F0 response relative to the original version, suggesting that the effect reflects intrinsic acoustic differences between voices rather than talker gender. Similarly, in another study, matching the pitch range of male and female narration resulted in comparable FFR amplitude and latency^50^. However, additional evidence indicated that FFR F0 representation is influenced not only by F0 but also by the harmonic distribution and formant structure of male and female voices. Male voices tend to provide more resolved low-frequency harmonics, whereas female voices rely more on higher-frequency components to generate the F0 response^23^. Together, these findings suggested that F0 representation reflects vocal acoustic properties rather than talker gender. Our findings further demonstrated that this acoustic dependence persists during dynamic tracking of emotional F0 contours.

Within the present sample, our results also revealed broadly similar F0 tracking between male and female listeners across conditions. Participants showed comparable performance in tracking prosodic F0 contours of the word “balloon” across emotions and talker voices. Although isolated differences appeared in RMSE for certain stimuli, the complementary 5% accuracy measure remained comparable, suggesting similar dynamic F0 tracking between male and female listeners. In line with this, Krizman et al. found that sex differences in the FFR to a /da/ syllable were limited to transient and fast components of the response, whereas F0 encoding, measured by both its spectral magnitude and the timing of F0-related peaks, was comparable across males and females. Our findings extend this pattern beyond overall F0 representation to dynamic tracking ability, as our analysis examined how accurately the central auditory system followed the time-varying F0 contour, rather than only the strength of phase-locked activity within the F0 band. In contrast, Ahadi et al. reported sex-related differences in the speech-evoked FFR using the same 40-ms synthesized /da/ syllable employed by Krizman et al., with females showing larger spectral representation of F0 and formant components as well as transient responses^24^. However, their analysis, like that of Krizman et al., was based on amplitude and spectral measures of phase-locking and did not assess the accuracy of F0 contour tracking. Another possible explanation for the observed differences is the type of speech material used. In the present study, listeners heard a meaningful and familiar word spoken with emotional intonation, whereas earlier studies used brief and isolated synthesized syllables. Previous work has shown that the FFR can vary with speech familiarity and even differs between speech and non-speech stimuli^51,52^. It is therefore plausible that sex-related differences reported for isolated syllables become less pronounced when listeners process more complex and rich speech. However, given the limited sample size, the study was not specifically powered to detect listener-sex effects, and replication in a larger sample is needed.

In conclusion, this study showed that the time-varying F0 changes in emotional speech can be extracted and tracked from FFR with good accuracy. However, the acoustic characteristics of the eliciting stimulus, including emotion- and talker-related pitch information can influence the ability of the FFR to track variations in F0 of the stimulus. Stimuli characterized by a lower F0, smaller F0 slope magnitude, and greater energy at F0 and its harmonics, such as sad emotion or male voices, can result in more robust F0 tracking in the FFR.

However, this study presents some limitations that highlight the need for further investigation and guide future research directions. While happy and sad emotions express very distinct variations in F0 contour, using a broader range of emotions in future works would allow for a more comprehensive investigation of how accurate the FFR can track various F0 contours. Also, we only used one male and one female speaker, making it unclear whether the observed effects of male/female acoustic features on the FFR, such as lower F0 and harmonics, can be generalized to other speakers. Nevertheless, despite the restricted stimulus set used, this work establishes baseline results for future studies on tracking neural responses to F0 changes in emotional stimuli. In addition, the use of isolated word “balloon” in repetition does not fully capture the complexity of emotional prosody in continuous speech. Building on recent work^22,53^, future studies using ecologically more valid longer stimuli, and control over listener engagement during the task, may provide further insight into the auditory processing of F0 dynamic cues in emotional speech in real-world listening contexts. Moreover, while this study focused on the F0 contour, future research could explore the neural encoding of other acoustic cues important for emotional prosody such as the intensity contour. Additionally, because the neural generators of the FFR remain debated^18^ and the present recording configuration does not permit source dissociation, further studies using more extensive recording configurations may investigate this issue. Future work is needed to extend this test to individuals with hearing impairment, with or without hearing aids. This would introduce speech prosodic assessment in clinical settings and thus provide a more comprehensive rehabilitation approach.

## Supplementary Information

Below is the link to the electronic supplementary material.

Supplementary Material 1

## Data Availability

Data will be made available on request.
