# Supplementary material for: Evaluating neural encoding of prosody-related F0 changes in emotional speech using the speech FFR in normal-hearing adults
Source: Sci Rep. 2026 Apr 29;16:19977. doi: 10.1038/s41598-026-50121-0 (PMC13319747; doi:10.1038/s41598-026-50121-0)
Supplement: Supplementary file 1 — Supplementary Material 1 [file 41598_2026_50121_MOESM1_ESM.docx]

Participant 1 (F)


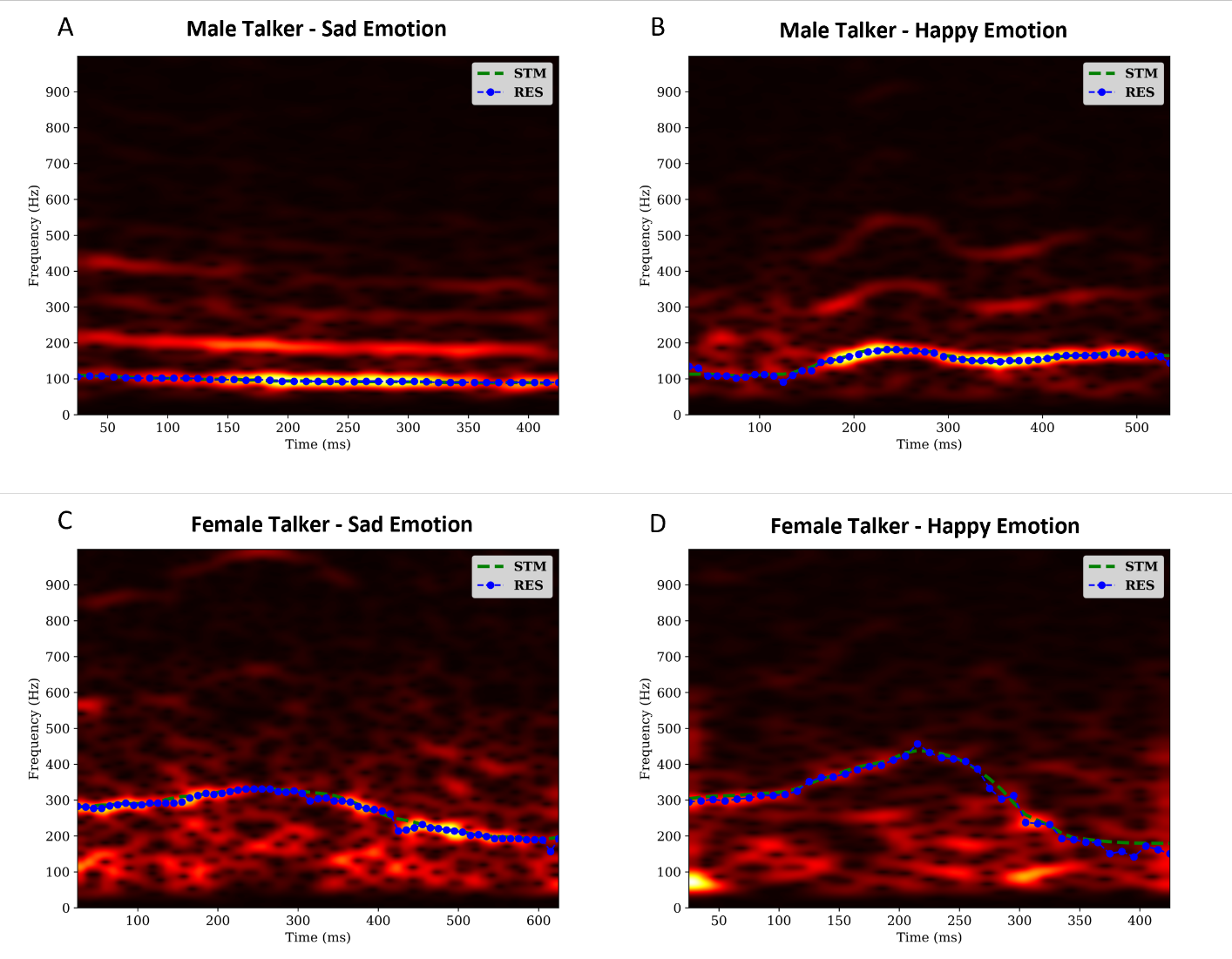


Participant 2 (F)


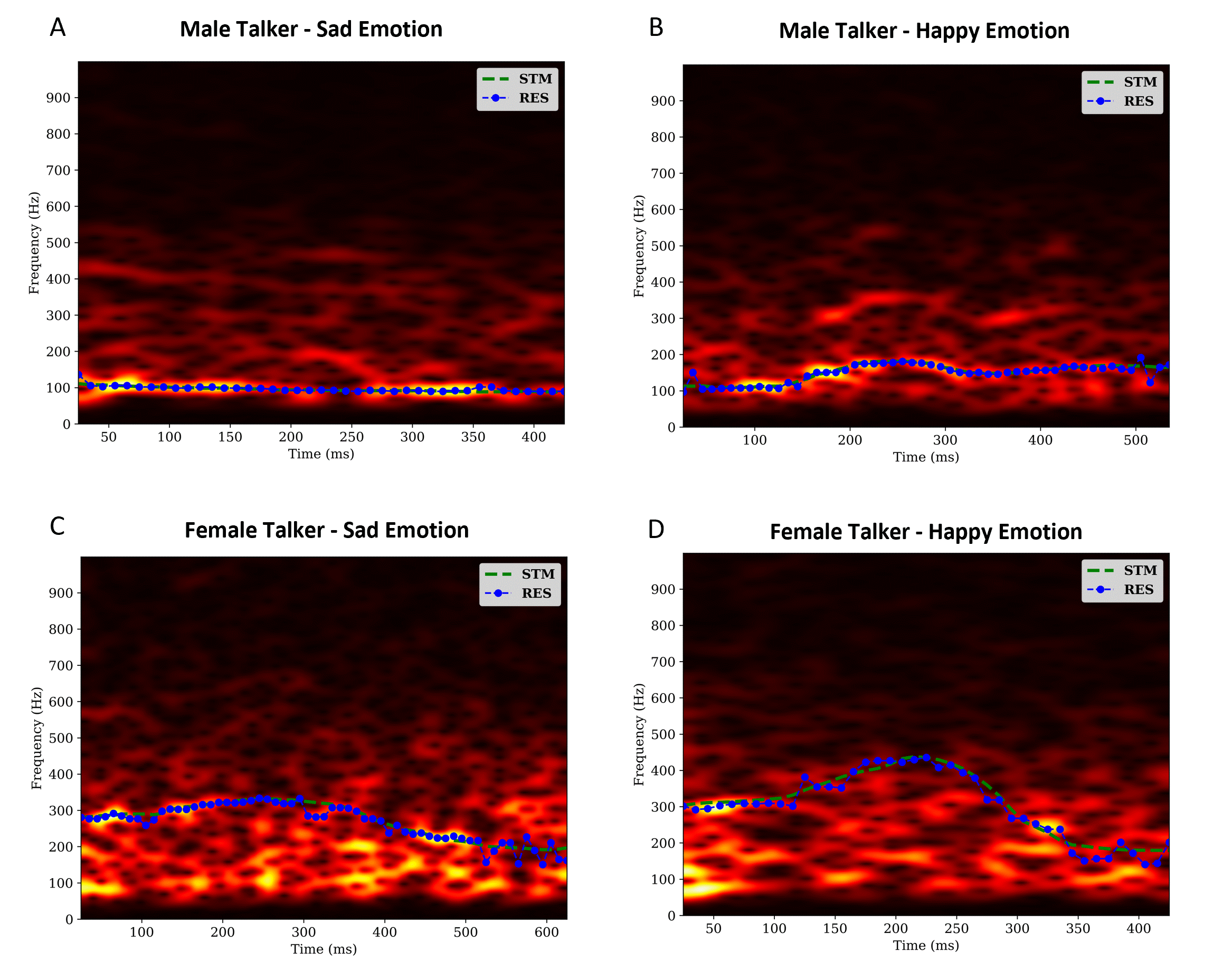


Participant 3 (M)


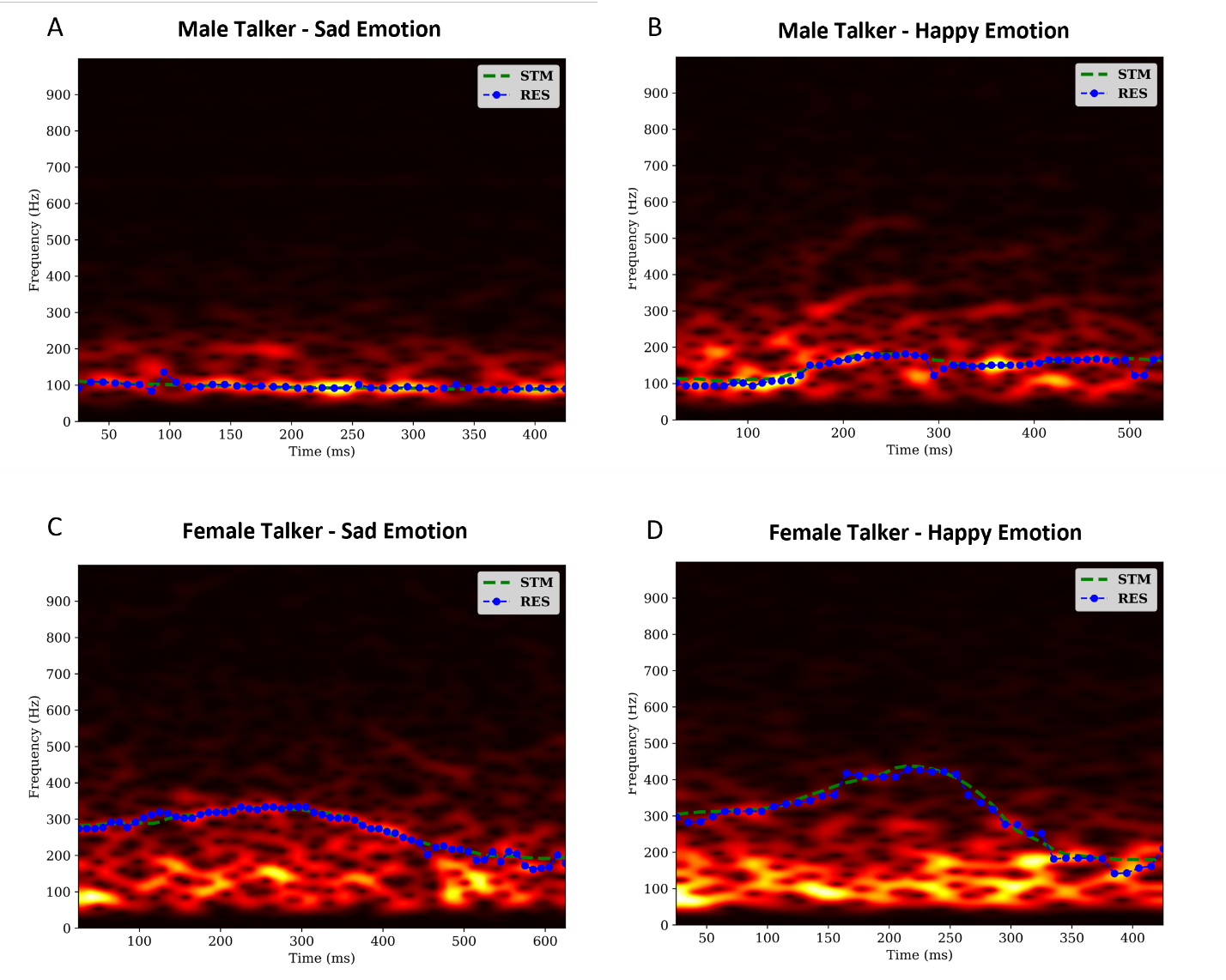


Participant 4 (F)


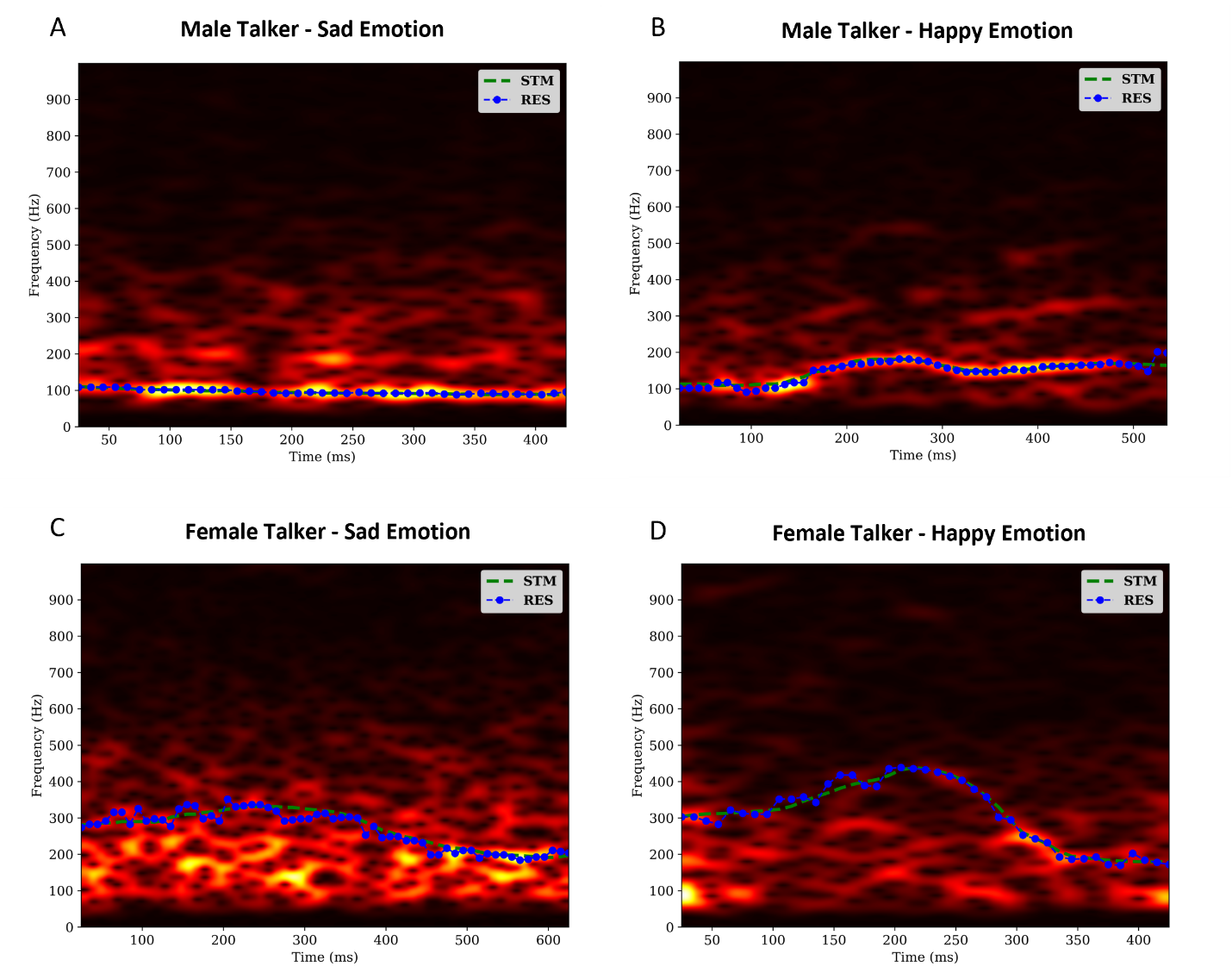


Participant 5 (F)


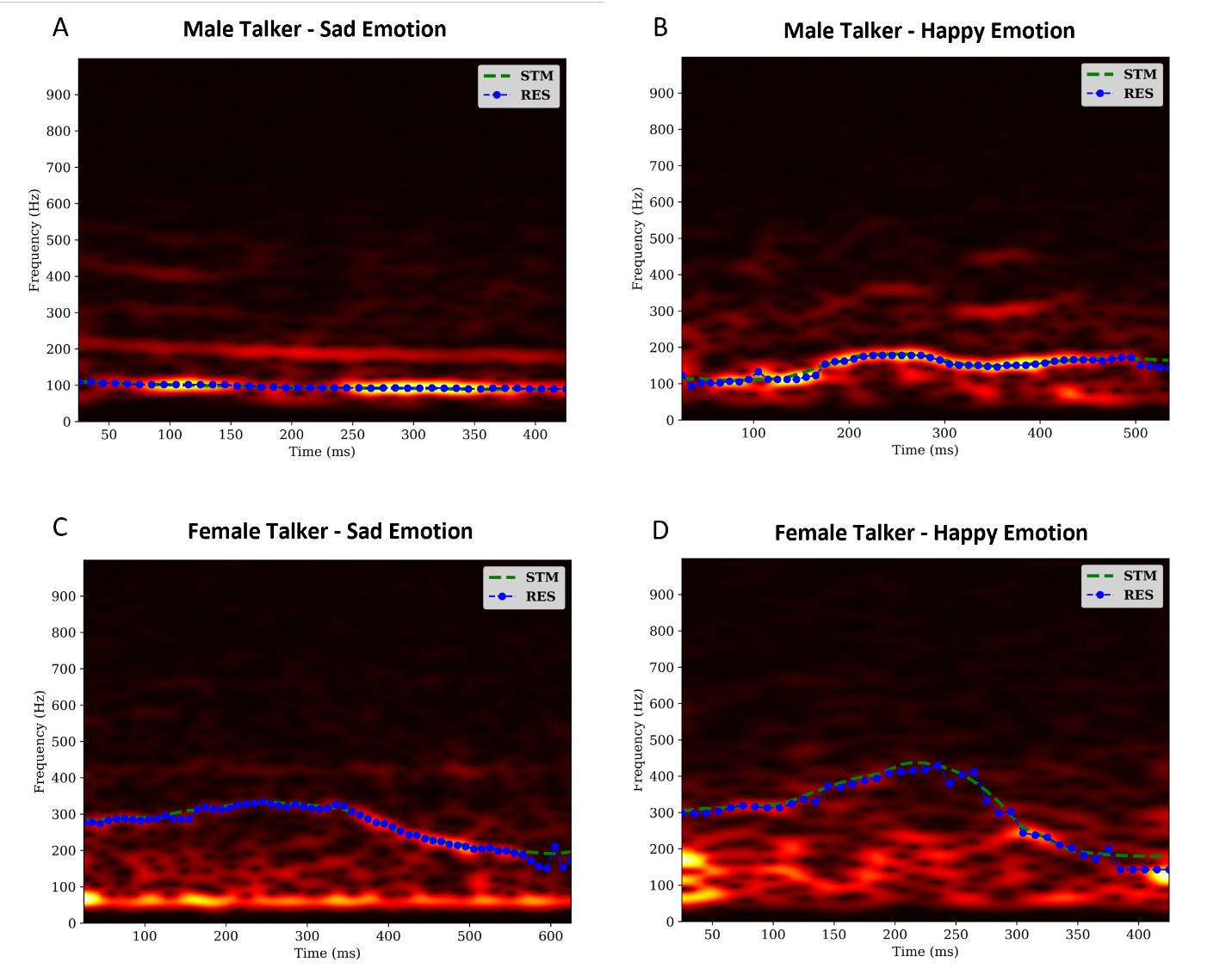


Participant 6 (M)


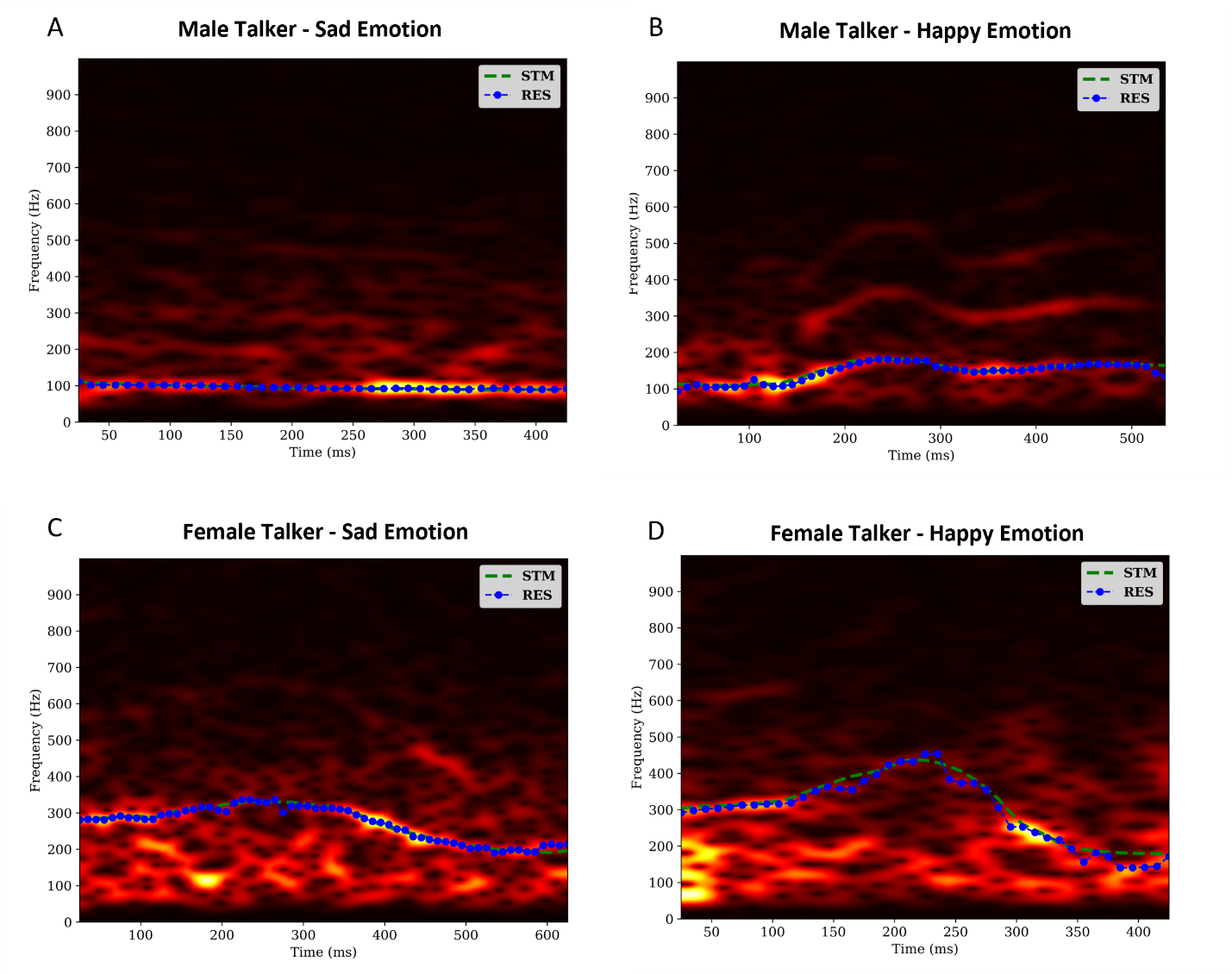


Participant 7 (F)


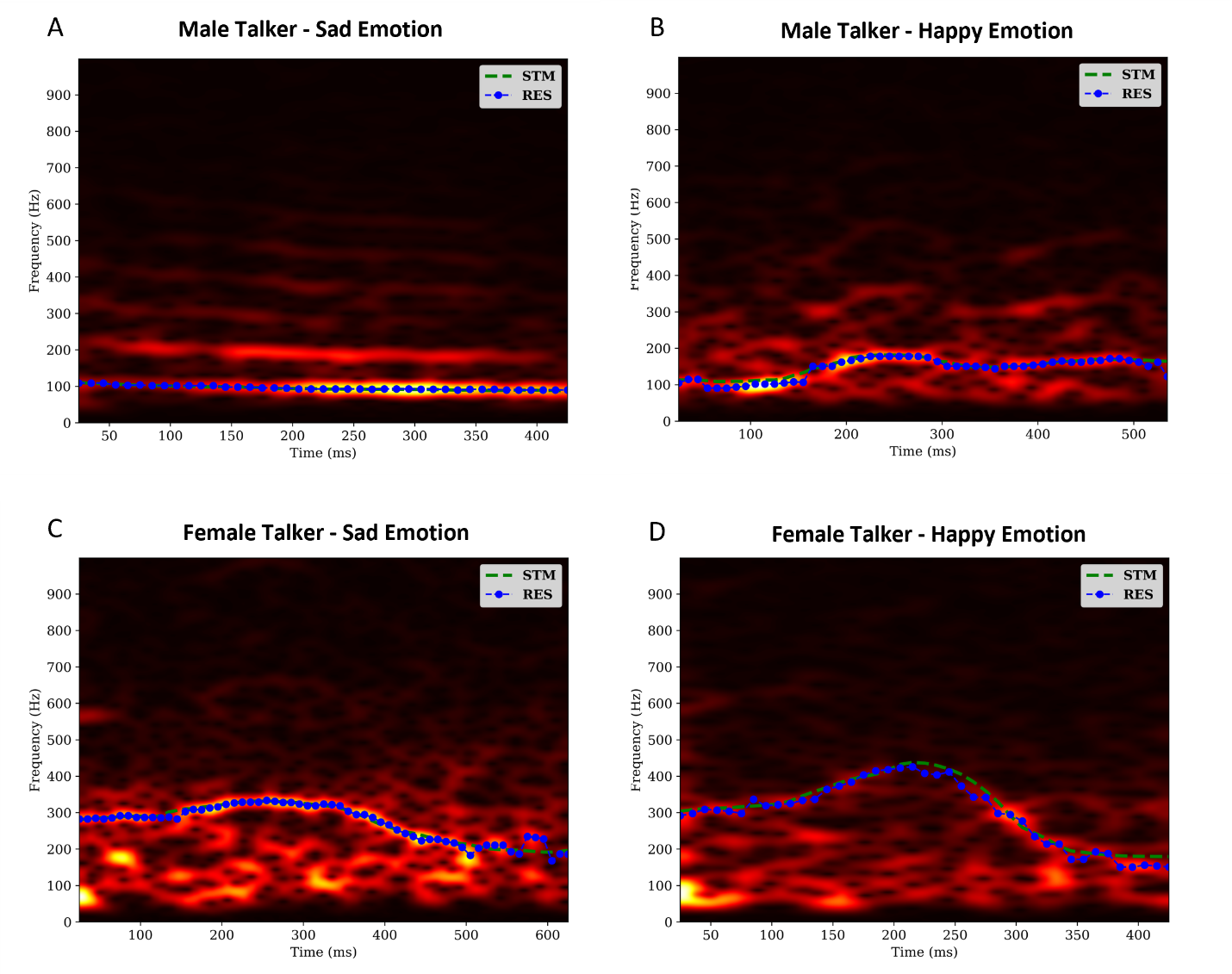


Participant 8 (F)


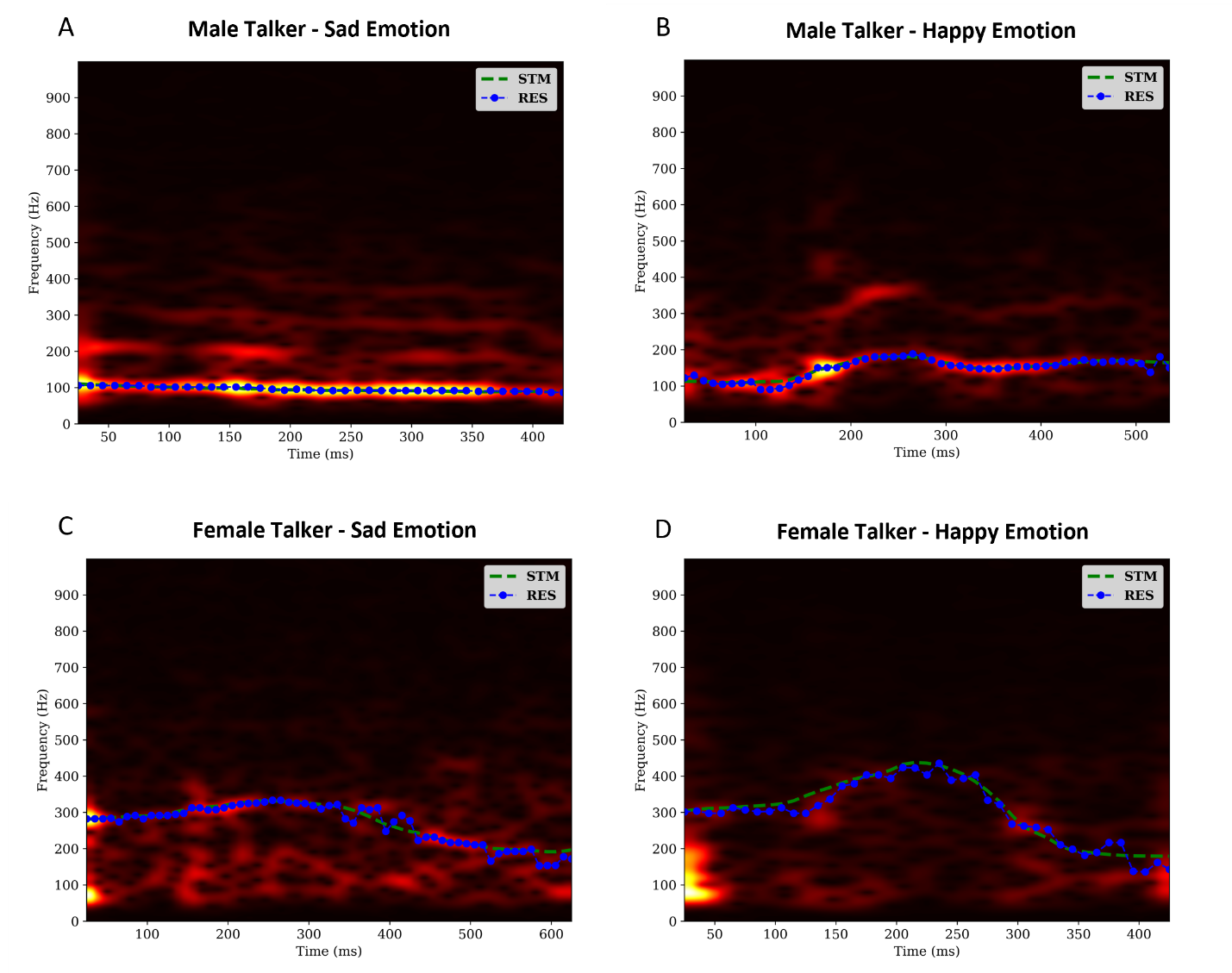


Participant 9 (M)


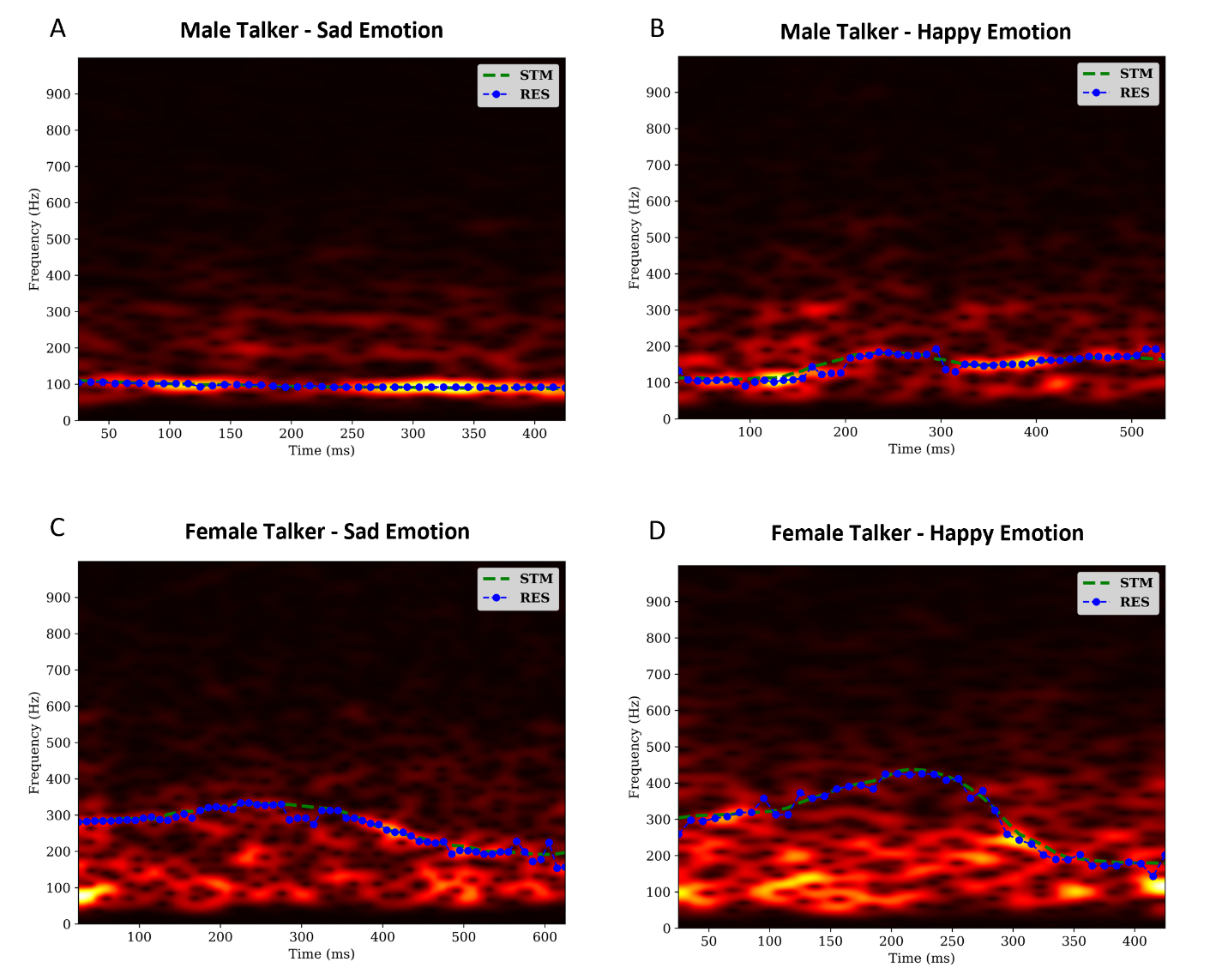


Participant 10 (M)


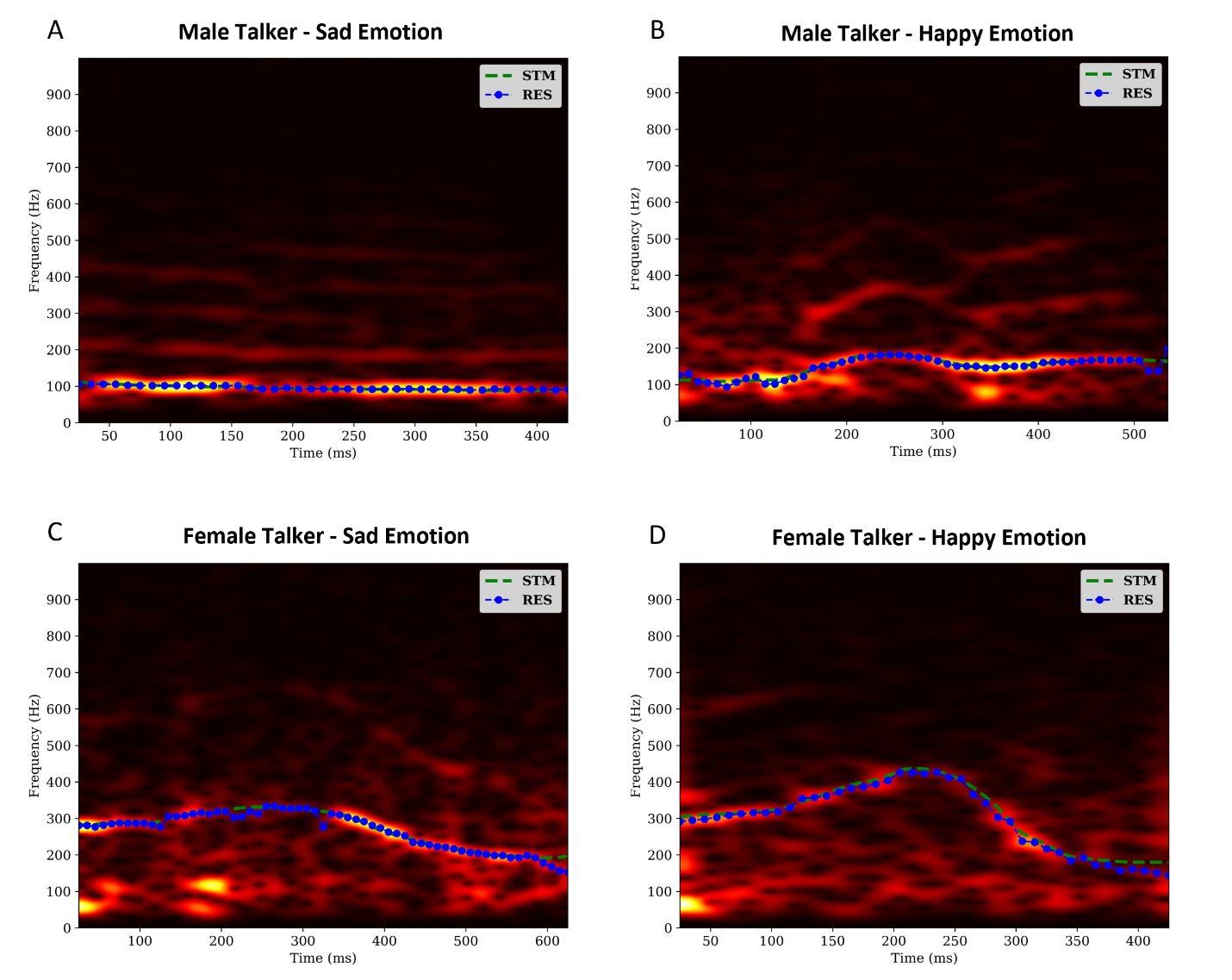


Participant 11 (M)


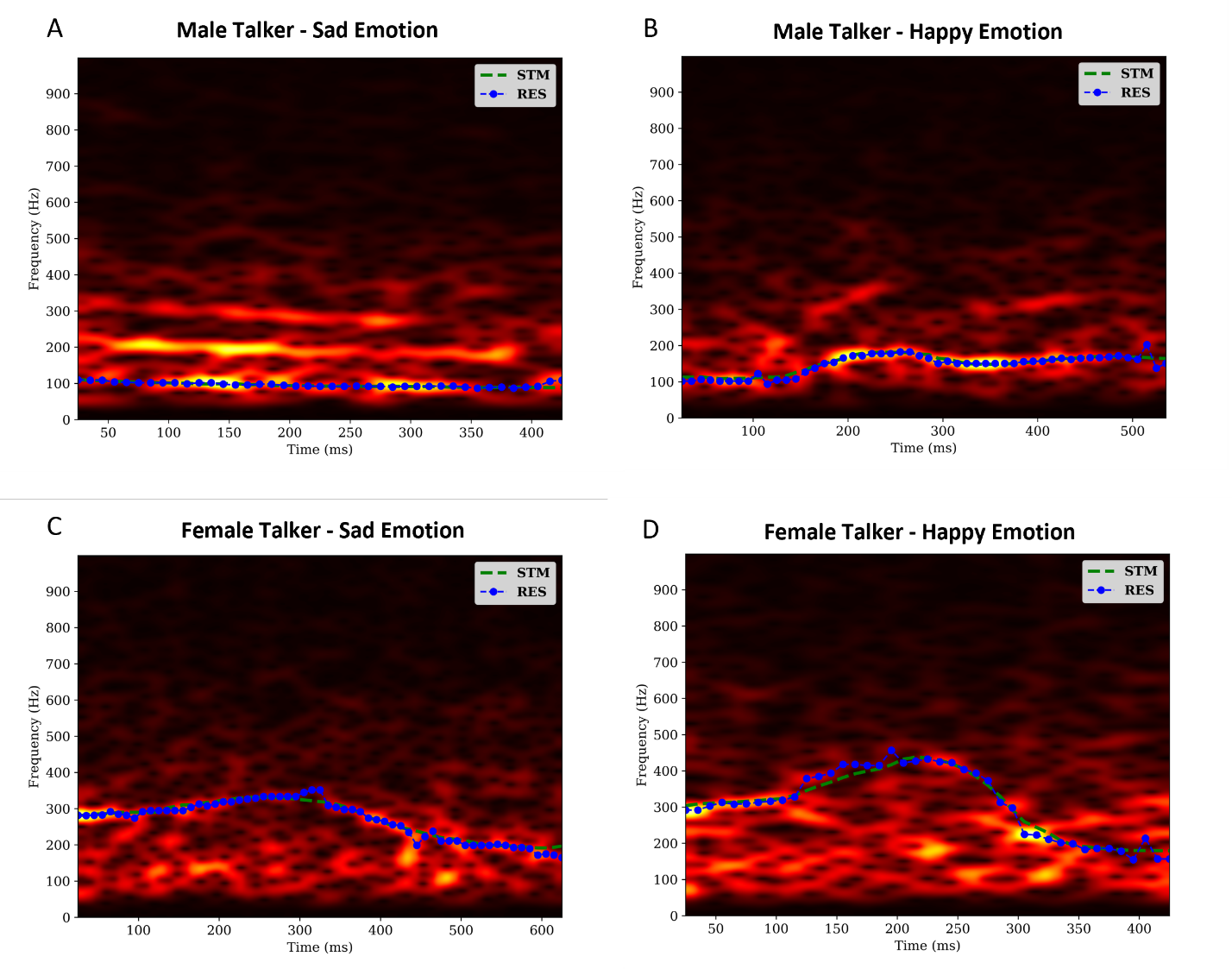


Participant 12 (F)


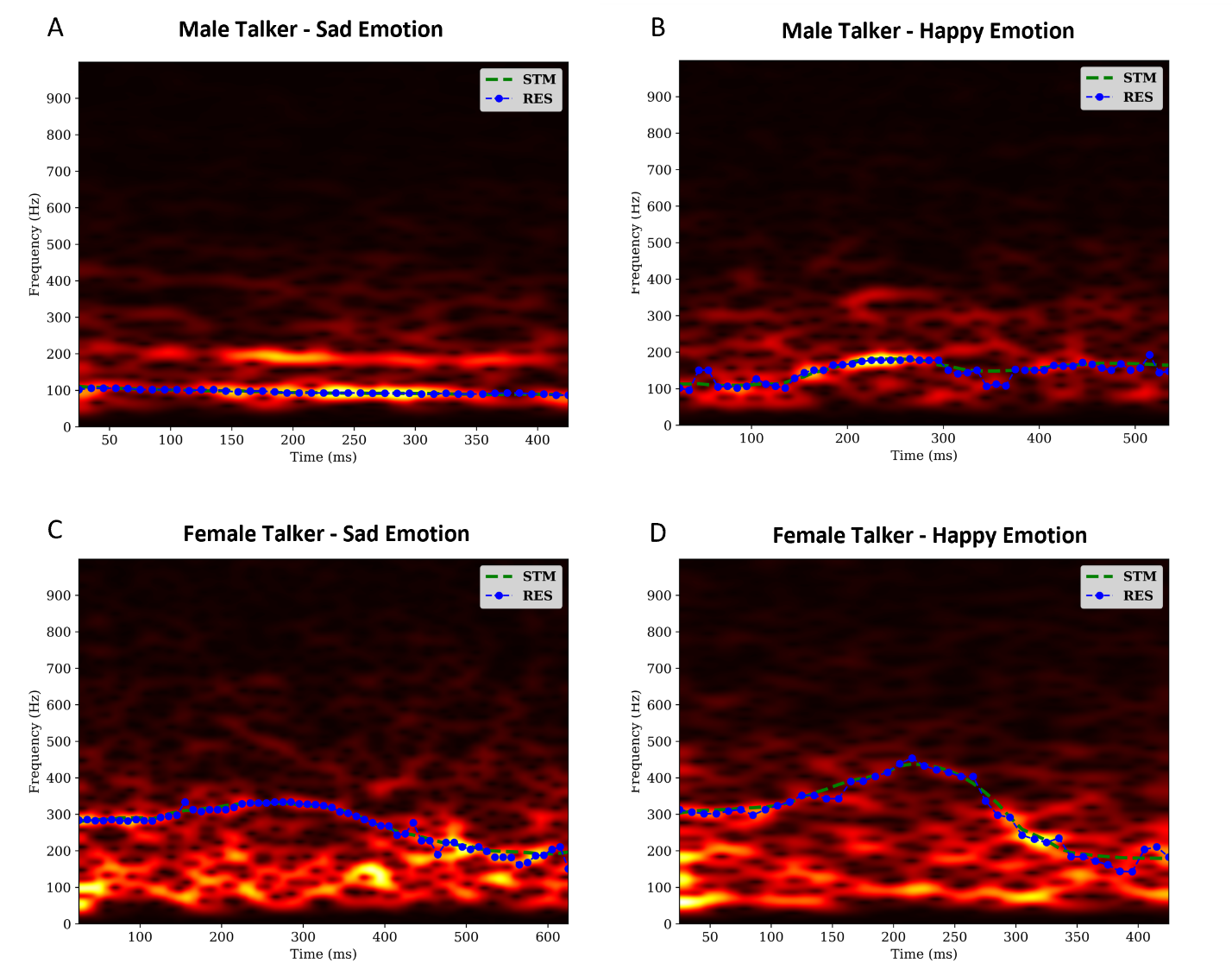


Participant 13 (M)


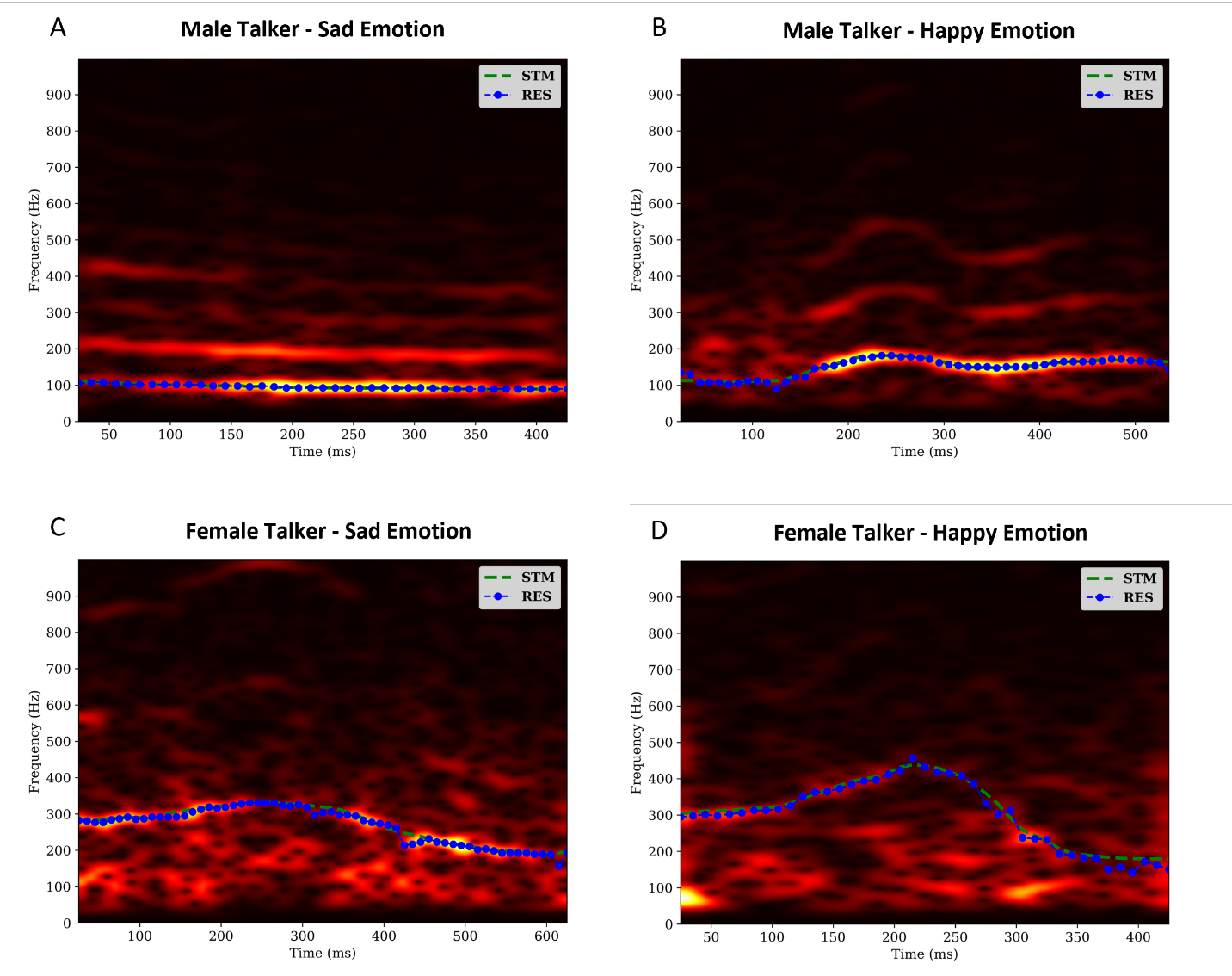


Participant 14 (F)


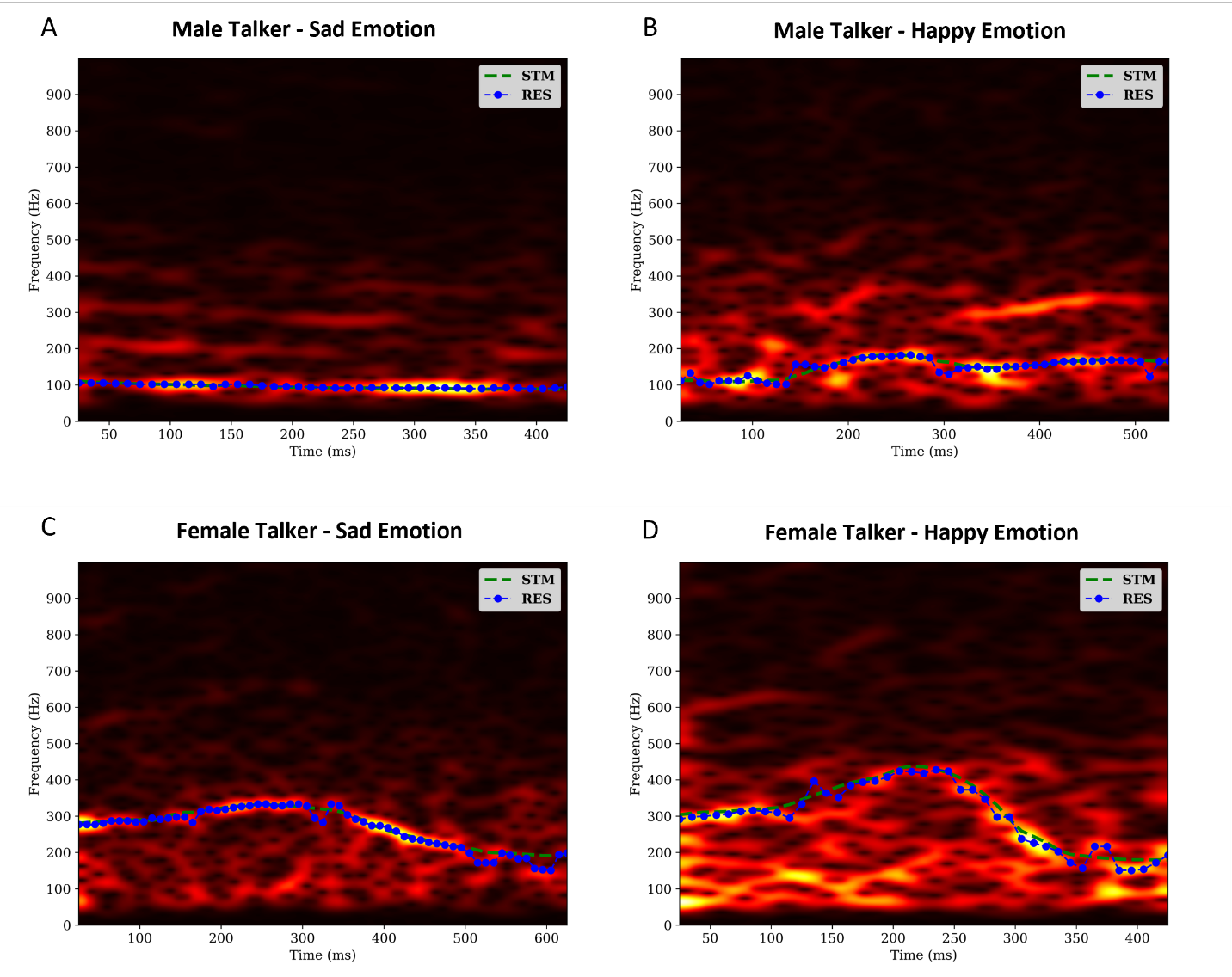


Participant 15 (M)


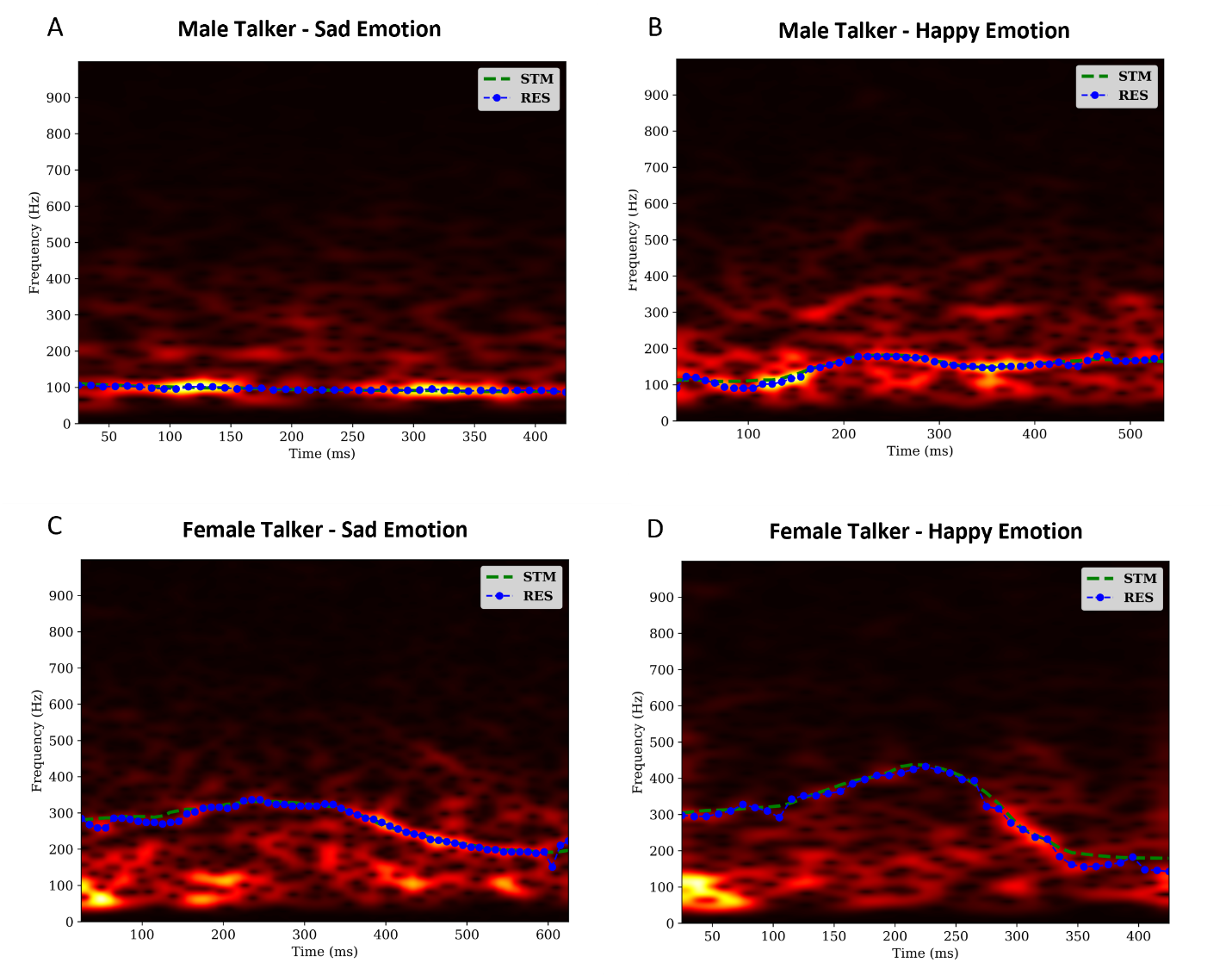


Participant 16 (M)


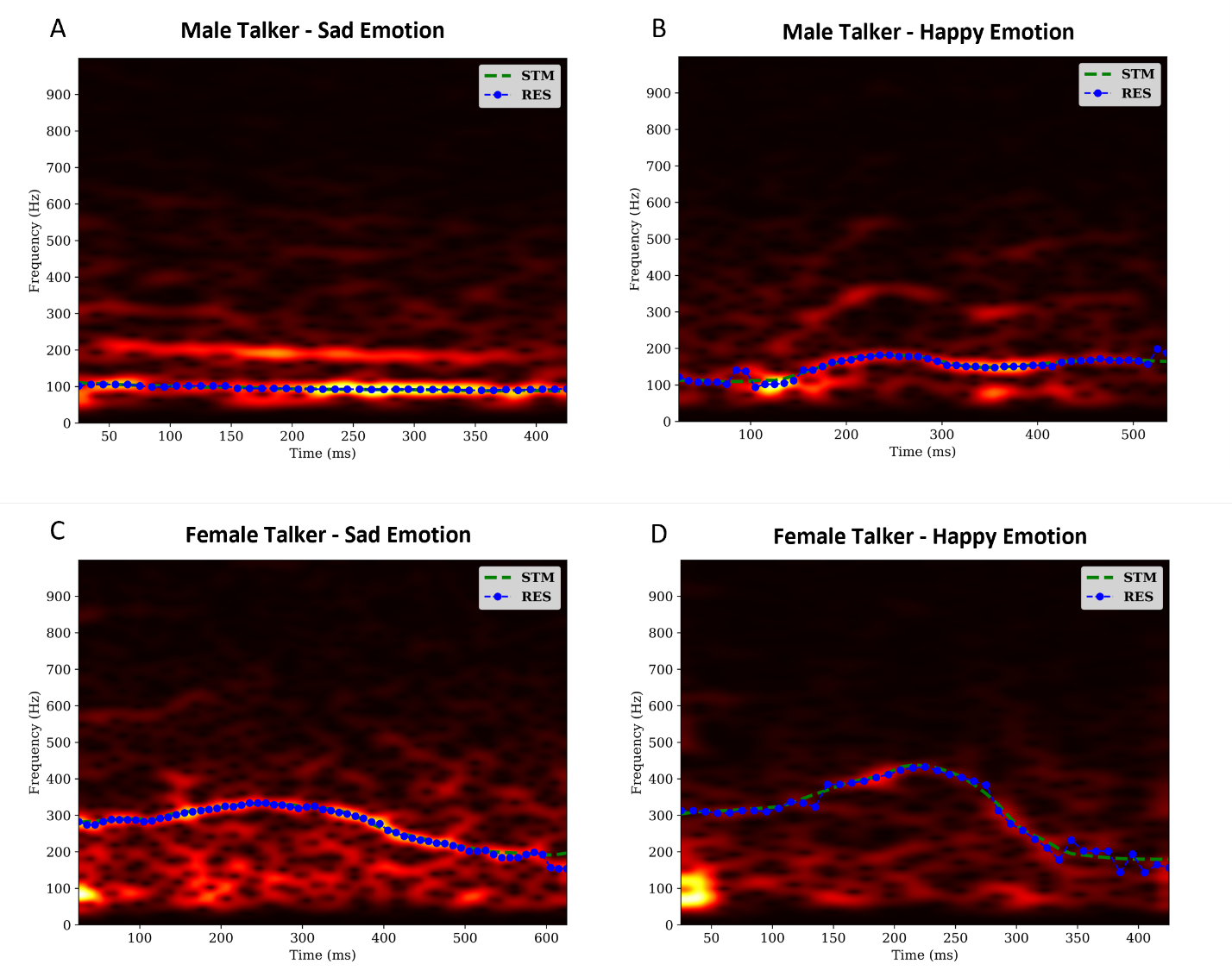


Figure S1. Spectrograms with overlaid F0 contours extracted from the stimulus waveform (green curves) and evoked FFR (blue curves) are shown for each participant (n=16) across the four experimental conditions: male talker and sad emotion (A), male talker and happy emotion (B), female talker and sad emotion (c), and female talker and happy emotion (D). Participant sex is indicated next to the participant number (F = female; M = male).
